# The Complex Relationship between Mesenteric Panniculitis and Malignancy — A Holistic Approach is Still Needed to Understand the Diagnostic Uncertainties

**DOI:** 10.7759/cureus.5569

**Published:** 2019-09-05

**Authors:** Veeraraghavan Meyyur Aravamudan, Shahab R Khan, Suresh Khanna Natarajan, Ikram Hussain

**Affiliations:** 1 Internal Medicine, Woodlands Health Campus, Singapore, SGP; 2 Internal Medicine, Banner University Medical Center, University of Arizona, Tucson, USA; 3 General Surgery, Khoo Teck Puat Hospital, Singapore, SGP; 4 Internal Medicine: Gastroenterology, Woodlands Health Campus, Singapore, SGP

**Keywords:** mesenteric panniculitis, malignancy, lymphomas

## Abstract

Mesenteric panniculitis is an idiopathic, localized inflammation involving the adipose tissue of the small bowel mesentery. The association of mesenteric panniculitis with malignancy, predominantly lymphomas, has been widely reported in the medical literature. In this review article, we will discuss the clinical guidelines in the diagnosis and management of mesenteric panniculitis and the clinical association between mesenteric panniculitis and malignancies.

## Introduction and background

Mesenteric panniculitis (MP) is an idiopathic, localized inflammation involving the adipose tissue of the small bowel mesentery. The association of mesenteric panniculitis with malignancy, predominantly lymphomas, has been widely reported in the medical literature. The majority of patients with mesenteric panniculitis are asymptomatic and are picked up incidentally while performing radiological examinations, but MP patients with a high risk of malignancy warrant a thorough investigation [1]. There is a lack of clear clinical guidelines on the management and follow-up of MP.

Cardinal radiological signs of mesenteric panniculitis

Table 1 shows the five cardinal radiological signs of MP [1].

**Table 1:** Five cardinal radiological signs of MP MP: Mesenteric panniculitis

#	Five Cardinal Radiological Signs of MP on Computed Tomography (CT) Scan
1	Fatty mass lesion in the small bowel mesentery
2	Hyper-attenuation of the mesenteric fat
3	Lymph nodes in the fatty mass
4	Halo surrounding the lymph nodes or vessels
5	Pseudo-capsule

Pathology

MP is an inflammatory disorder of the mesenteric root with two distinct pathological subgroups: mesenteric panniculitis and retractile mesenteritis. The differential diagnosis of these two conditions is based on histological criteria; fat necrosis predominates in MP whereas fibrosis and retraction predominate in retractile mesenteritis [2].

The exact diagnosis is often difficult. It is usually made by finding one of three major pathological features: fibrosis, chronic inflammation, or fatty infiltration of the mesentery.

Clinical features

The majority of patients with mesenteric panniculitis are asymptomatic, although some may present with non-specific symptoms like abdominal pain, nausea, vomiting, fever, ascites, and pleural effusion [2].

The condition occurs mostly in middle or late adulthood with a slight male predominance. Symptoms may be progressive, intermittent, or absent. Laboratory findings, including elevation in erythrocyte sedimentation rate and anaemia, are generally absent or non-specific.

Aetiology

Common causes of MP include abdominal trauma and a history of abdominal surgery. Associated inflammatory disorders, such as vasculitis or chronic rheumatic conditions, granulomatous disease, rheumatic disease, malignancies, and pancreatitis have also been reported.

Infectious associations with MP include mycobacterial and cryptococcal infections and cholera. In some patients, especially those having an acute presentation of the disease, viral mesenteritis is likely. Fever of unknown origin and chylous ascites have also been described in patients with MP [3].

Although often entirely isolated, synchronous association of MP has been observed with some neoplastic diseases, such as lymphoma, colorectal cancer, and melanomas. The possibility of MP being a paraneoplastic syndrome in the elderly should be considered [2].

When MP occurs in association with malignancy, the most common primary sites are the large bowel, the lymph nodes, and the urogenital tract. In those with MP, any cancer - with the exception of prostate cancer - can usually be seen on an index computed tomography (CT) scan. Further extensive investigation in asymptomatic patients is therefore likely to be of low yield [4].

## Review

Objectives of this literature review

To discuss the clinical features and association between mesenteric panniculitis and malignancy, the diagnostic dilemmas, and their treatment plan.

Materials and methods

We conducted a literature search of articles using the US National Library of Medicine PubMed database, PubMed, MEDLINE, Embase, Cochrane Library and Google Scholar databases, ClinicalTrials.gov for studies, and the ISI Web of Science. No date restrictions were placed on the search. A thorough search for controlled clinical trials and cohort studies was conducted. Since the rarity of condition, case reports were also included.

We used the keywords: "Mesenteric panniculitis" and "malignancy".

Included studies were those published in English that assessed the association between mesenteric panniculitis and malignancy. Reference lists were also screened. From the search results, articles with irrelevant titles were discarded, with the remaining abstracts examined for relevance.

The authors of this review independently determined the eligibility of the studies and assessed the methodology of included studies. In this review article, we will discuss the aetiology, pathogenesis, and clinical studies related to MP, as well as case studies and their management per the latest clinical guidelines.

Review of clinical studies

The findings of the literature review are summarised in Table 2, but we will discuss a few of the studies that are more relevant to the association between mesenteric panniculitis and malignancy.

**Table 2:** Summary of clinical studies

Study Author(s)	PubMed ID	Findings
Akram et al. [5]	17478346	Patients with symptomatic MP may benefit from a combination of tamoxifen and prednisolone.
Al-omari et al. [1]	30643446	A study of 116 MP patients diagnosed by CT scan showed that those with a greater diameter of the MP mass were more than twice as likely to also have malignancy.
Badet et al. [2]	25701479	In 158 patients with MP (diagnosed via radiology), neoplasia was present in 88, including 25 with lymphoma, 16 with melanoma, and 13 with colorectal cancer.
Béchade et al. [6]	17316921	Out of seven MP patients, four also had a diagnosis of breast cancer, non-Hodgkin's lymphoma based on peripheral lymph node biopsy and cryptoglobulinemic vasculitis based on renal biopsy.
Buchwald et al. [7]	27515476	Out of 173 patients with possible MP, 43% (75) were diagnosed with malignancy.
Coulier et al. [8]	22191288	Of the 48 patients with a diagnosis of MP, 7 patients were eventually diagnosed with malignancy.
Cross et al. [4]	26467030	Out of 259 patients with confirmed MP, 78 were diagnosed with malignancy (54 with a current cancer and 33 with a past cancer or both); the most common primary sites were colorectum (19), lymph nodes (17), kidney (6), and prostate (4).
Daskalogiannaki [9]	10658720	CT evidence of MP was observed in 49 patients. MP coexisted with malignancy in 34 patients, and it coexisted with benign disorders in 11 patients.
Ehrenpreis et al. [3]	28082812	A total of 359 patients had CT scans with signs of MP-like abnormalities; 81 patients had a known history of cancer at the time and 19 had a new cancer diagnosis at the time of their CT. Fourteen of these patients were undergoing CT as part of a malignancy evaluation. The most common cancer associated with MP-like signs on the CT was lymphoma with 36 cases (17 of which were follicular lymphoma).
Gögebakan et al. [10]	23906444	Out of 13,485 CT patients, 77 were diagnosed with MP; of these, 51% were also diagnosed with malignancy vs. 60% of the control group (those without MP).
Khasminsky et al. [11]	28712750	Among MP patients, 1.8% were found to have NHL, which is about how prevalent it is in the general population.
Küpeli et al. [12]	29914254	Out of the 22,033 patients in this study, 309 were diagnosed with MP; 58% of them also had a malignancy.
Mahafza et al. [13]	28917065	Of the 4,758 patients in the study who underwent abdomino-pelvic MDCT, 90 patients had MP-like features. Twenty-eight of those patients were also diagnosed with malignancy, which represented a risk more than two times higher than for those without MP.
Sahin et al. [14]	29073610	Of the 19,869 CT scans, 36 patients had MP. Twenty-four of them were categorized as idiopathic, and malignancy was the predisposing factor in 8 of those patients.
Van Putte-Katier et al. [15]	25271412	Consecutive abdominal CT examinations of 3820 patients were evaluated for MP. Clinical characteristics, therapy and outcome of patients with MP were evaluated during a 5-year follow-up period. Ninety-four (2.5%) patients with MP were identified (mean age, 66.6 ± 11.2 years, 70.2% male). MP coexisted with malignancy (especially prostatic carcinoma) in 48.9% of patients, and this was slightly but significantly higher than in age- and sex-matched control patients (n = 188, 46.3%). In 48 patients, MP was presumed to be idiopathic.
Scheer et al. [16]	27529397	Retrospective analysis of consecutive CT abdomen examinations of 5595 patients in terms of MP over a period of 3 years was performed. A total of 143 cases were diagnosed with MP (2.55 %). The average age of patients was 69.9 years with a male to female ratio of 2:1. In this group oncological disease was confirmed in 107 patients (74.8 %). In 36 patients with MP (25.2 %), no malignancy was present. In the group of patients with an underlying oncological disease, the prevalence of MP was 5.42 % and was significantly higher (p
Protin-Catteau et al. [17]	26868171	Retrospective search for MP reviewing 3054 consecutive multidetector row computed tomography (MDCT) scans. Two radiologists in consensus selected the final MP population. For each MP, two subsequent MDCT scans of patients matched by gender and age. Five-year follow-up data regarding cancer occurrence after index MDCT scans were obtained for the MP and control groups. Comparisons between groups were performed using univariate conditional logistic regression. Results: A total of 160 patients had at least three of the five MDCT features defining MP. Sixty-four were excluded owing to disease causing mesenteric infiltration or contiguous neoplastic involvement. The final population included 96 MP and 192 control patients. The prevalence of MP was 3.14%. Most cases of MP were discrete (66.7%), 2.1% were marked. In total, 60.4% and 59.4% of MP and control patients, respectively, had cancer (p = 0.86). There was no significant association between MP score and presence of cancer (p = 0.06) nor any relationship between the course of associated cancer and MP evolution. In total, 80/96 MP patients and 50/78 control patients without associated cancer had a 5-year follow-up at least. No significant difference between both groups for new tumor occurrence during follow-up was found (p = 0.15). Results do not suggest that patients with incidentally found MP should be followed up for early detection of a cancer.
Smith et al. [18]	22706134	Three hundred fifty-nine patients were identified, 81 (22.6%) had a known malignancy at the time of the index abdominal CT scan. Nineteen (6.8%) of the 278 had a new diagnosis of malignancy on evaluation of the findings of the index CT scan. Among the 240 (86.33%) that did not have a notation of the abnormality in their medical record, 11 (4.58%) developed a malignancy during the study period. Sixty-eight of the 248 (24.46%) without a known malignancy had diseases associated with mesenteric abnormalities. The presence of these were associated with a reduction in the likelihood that the abnormalities are associated with new or delayed diagnosis of a malignancy (odds ratio, 0.197; 95% confidence interval, 0.0045-0.8501; p = 0.013). Progression of underlying malignancy was unlikely in those where the mesenteric abnormalities did not worsen in appearance on follow-up CT scans (odds ratio, 0.03268; 95% confidence interval, 0.0028-0.3761; p = 0.0061). In the presence of an underlying disease associated with these findings, the subsequent finding of a malignancy is less likely. In addition, neglect of these findings may result in delayed diagnosis of cancer.
Wilkes et al. [19]	22706134	One hundred eighteen (92 males; median age, 61 years; range, 20-88 years) patients were identified with mesenteric panniculitis. Malignancy was identified in 45 patients (38%) (34 males). The most common malignancies were colorectal (14), lymphoma (13), and urogenital tract (7). Malignancies were diagnosed after the detection of mesenteric panniculitis in 13 patients. Univariate analysis of demographic, clinical, and radiological features revealed that lymph node size >12 mm (relative risk 4.5 (CI 1.4-14.6); p = 0.0266) and the absence of the fat ring sign (relative risk 0.6 (0.3-1.1); p = 0.047) were associated with the subsequent diagnosis of malignancy in patients with mesenteric panniculitis.

The authors of this review gathered all the data showing the relationship between mesenteric panniculitis and malignancy. Various variables like study design, age, gender, total number of patients with malignancy, colorectal cancer, pancreatic cancer, lymphoma, cholangiocarcinoma, prostate cancer, breast cancer, bladder cancer, lung cancer, metastases during follow-up, previous abdominal surgery, inflammatory bowel disease, autoimmune disease, and death are gathered and discussed in Table 3. In particular, mesenteric panniculitis and colon cancer are also discussed.

**Table 3:** Demography of clinical studies NR: Not reported

Author	Pubmed ID	Study design	Age	M/F		Total patients (n)		Patient with malignancy		Colorectal cancer		Pancreatic cancer		Lymphoma		Cholangio-carcinoma		Prostate cancer		Breast cancer		Bladder cancer		Lung cancer		Metastases during followup		Previous abdominal surgery	Death		Inflammatory bowel disease		Autoimmune
Al-Omari et al. [1]	30643446	Retrospective, January 2014 to January 2017, Single center, Jordon. // Group 1 (n = 73) - Without primary malignancy	54.29 ± 13.03	45/28		73		0		NR		NR		NR		NR		NR		NR		NR		NR		NR		NR	NR		NR		NR
		Group 2 (n = 43) - With primary malignancy	64.77 ± 11.41	24/19		43		43		6		1		8		1		2		10		3		4		NR		NR	NR		NR		NR
Ehrenpreis et al. [3]	28082812	Retrospective, January 2005 to April 2010, Multicenter, Chicago, USA	NR	NR		359		81 known cases, 19 new cases		6		NR		36		NR		7		4		5		6		NR		NR	NR		NR		NR
Kaya et al. [20]	30023976	Retrospective, January 2010 to March 2016, Single center, Istanbul, Turkey	45.8 ± 15.7 years	17/5		22		4		1		NR		NR		NR		2		NR		NR		NR		NR		NR	NR		NR		NR
Mahafza et al. [13]	28917065	Retrospective, January 2012 to December 2014, Jordan University Hospital, Amman, Jordan	Males (mean age ± SD = 61.6 ± 15.3 years; range, 21-92); females (mean age ± SD = 62.8 ± 16.7 years; range, 38-84)	41/49		90		28		7		2		3		NR		3		6		1		1		NR		44	NR		NR		NR
Nyberg et al. [21]	28610559	Retrospective, 2005-2012, Multicenter, Stockholm	Median age at diagnosis was 50 (IQR 44; 72) years	16/11		27		NR		NR		NR		NR		NR		NR		NR		NR		NR		NR		3	NR		NR		NR
Sahin et al. [14]	29073610	Retrospective, January 2012 to December 2015, Turkey.	54 years (range 26 – 76)	17/19		36		8		2		2		NA		NR		NR		1		1		NR		NR		3	NR		NR		NR
Badet et al. [2]	25701479	Retrospective 2004-2013. France	63 years (27–98)	121/37		158		88		13		4		25		NR		11		4		1		3		NR		61	NR		NR		6
Canyigit et al. [22]	21882092	Retrospective, Dec 2007 to May 2009, Multicenter, Turkey.	33-78 yrs (mean 56.2 yrs) (of 51 patients)	NR		2100		9 (of 51)		NR		NR		NR		NR		NR		NR		NR		NR		NR		17	NR		NR		NR
Khasminsky et al. [11]	28712750	Retrospective, 2008-2013, Single center, Israel (NHL)	19-94 yrs (mean age 64.06 yrs)	113/53		3 out of 166 NHL patients had mesenteric panniculitis		166		NR		NR		166		NR		NR		NR		NR		NR		NR		NR	NR		NR		NR
		(Control)	42-84 yrs (mean 65.72)	226/106		7 out of 332 in the control had mesenteric panniculitis		NR		NR		NR		NR		NR		NR		NR		NR		NR		NR		NR	NR		NR		NR
Buchwald et al. [7]	27515476	Retrospective, January 2003 - December 2015, Single Center, New Zealand	63 (range 27–90) yrs	131/42	173		75		NR		NR		NR		NR		NR		NR		NR		NR		NR		NR			NR		NR	NR
		(Control)	65.72 +/- 14.55 years	128/64	192		114		30		NR		14		NR		6		2		NR		10		NR		66			NR		4	4
Cross et al. [4]	26467030	Retrospective, January 2003-December 2014, Single center, New Zealand	60 yrs (20–94)	185/74	259		78		12 + 7		1		17		2		4		1		2		1		NR		NR			21		NR	NR
Gögebakan et al. [10]	23906444	Retrospective, January 2010 - October 2012, Single center, Netherlands (Mesenteric panniculitis)	65.5 ± 11.9 yrs	59/18	77		39		12		4		6		2		4		3		NR		9		NR		10			NR		NR	NR
		(Control)	66.0 ± 11.4 yrs	NR	152		93		26		3		23		17		11		2		NR		31		NR		33			NR		NR	NR

Although prior studies have described the association of MP and malignancy, a recent study shows that only 1.4% of patients with a computed tomography (CT) scan finding of MP will be found to have a previously undiagnosed or suspected cancer [3]. The higher rate of association of MP and cancer described in prior studies likely indicates the inclusion of patients with a known history of cancer.

Additionally, this study shows that a follow-up abdominal CT in patients with cancer suggests stability and not a worsening of MP. Finally, findings indicated that positron emission tomography scans are not recommended in the evaluation of cancer patients with mesenteric panniculitis-like findings on a CT [3].

One retrospective study of 4,758 patients with 90 identified cases of mesenteric panniculitis found that the likelihood of associated malignancy (mostly intra-abdominal malignancy) was 2.1 times higher in patients with MP than those without it [13].

The crude ratio of mesenteric panniculitis patients with colon cancer is less than 10% from our studies (refer Table 4), which is worth looking into. Bigger studies with good sample size and proper research are necessary to further assess it. Even though this is simply a crude ratio, it holds promise for better understanding of the co-occurrence of MP and colon cancer.

**Table 4:** Mesenteric panniculitis (MP) and colon cancer

Author	Total patients (n)	Colorectal cancer	Crude ratio (%)
Al-Omari et al.	43	6	13.95
Ehrenpreis et al.	359	6	1.67
Kaya et al.	22	1	4.54
Mahafza et al.	90	7	7.77
Van Putte-Katier et al.	94	8	8.51
Sahin et al.	36	2	5.55
Scheer et al.	143	20	13.98
Badet et al.	158	13	8.22
Protin-Catteau et al.	288	38	13.19
Cross et al.	259	19	7.33
Gogebakan et al.	229	12	5.24
Smith et al.	359	10	2.78
Wilkes et al.	118	14	11.86
Daskalogiannaki et al.	49	5	10.20

Follow-up

Computed tomography scan is optimal for accurate, non-invasive diagnosis of MP and follow-up of sclerosing mesenteritis and any complications. The presence of some radiological findings, such as lymph node size of more than 12 mm and the absence of the fat ring sign, should raise the concern of subsequent malignancy in patients with MP [23].

Treatment

There are no well-established treatment plans for this rare condition. Thus, any treatment prescribed is mainly for symptom relief and to address any complications. Commonly used agents include steroids and other immunosuppressants [24].

One study found that symptomatic patients with idiopathic mesenteric panniculitis responded to treatment with antibiotics and non-steroidal anti-inflammatory drugs (NSAIDs) [9]. Patients with obstructive or compressive symptoms may require surgery.

Strengths of the studies

This is one of the most comprehensive literature reviews discussing the association between mesenteric panniculitis and malignancy. The studies in this literature review have been done in multiple centres which will increase the generalisability of the results within a population. Studies also represent a wide range of malignancies like colorectal cancer, lymphoma, breast cancer, etc.

Limitation of clinical studies

Most clinical studies performed on MP lacked histological biopsies. Generally, a biopsy is not justified due to the incidental asymptomatic nature of the disease in most patients. For the majority of patients, the diagnosis was based on the CT appearance and on follow-up CT studies that revealed no additional findings or changes [6].

Most of studies are retrospective and details regarding standardisation of CT scan protocol like intravenous contrast and oral contrast were not available.

Various aspects of interest are not included such as race/ethnicity, medications, chemotherapy and are not discussed in detail.

Discussion

Mesenteric panniculitis is a rare clinical entity that can occur independently or in association with other disorders. Diagnosis of this nonspecific, benign inflammatory disease presents a challenge to gastroenterologists, radiologists, surgeons, and pathologists.

In most cases, MP is self-limiting and regression can even be observed during follow-up in the absence of medical treatment. Clinical symptoms can subside without surgery and with the use of drugs such as corticosteroids, colchicine, cyclophosphamide, and tamoxifen. MP is considered not to be precancerous, and hence long-term follow-up is not needed [20].

There is a lot of dilemma clinicians facing regarding follow-up CT imaging in patients with mesenteric panniculitis. The clinicians should also not subject the patient to unnecessary imaging which also puts the patients at increased risk of radiation-induced gastrointestinal malignancies. The main dilemma clinicians face after diagnosis of MP is how to follow it up and what should be the frequency of scanning. As such CT remains the most widely used and cost-effective modality for adult patients. The frequency of scanning should be guided by clinical symptoms and aetiology of MP. For MP associated with benign causes and in asymptomatic patients, frequency of scanning can be less, unless there is change in clinical symptoms. It would be practically prudent to suggest yearly follow-up CT at first instance but we need clinical guidelines and clinical studies to support this.

Radiological imaging like magnetic resonance imaging may be a reasonable option but it may be expensive and ultrasound may not be the best modality as it can miss findings which CT scan can identify. Ultrasound and magnetic resonance imaging can be used as follow-up modalities for paediatric patients and patients with renal impairment.

Histopathological confirmation is usually not needed to establish the diagnosis of MP as radiological features often suffice. Biopsy should be reserved for cases where there is suspicion of associated malignancy, for example in a scenario where follow-up CT scans are showing progressively enlarging mesenteric nodes on the background of MP, hence raising suspicion of lymphoma. Wait and watch approach can be used for MP secondary to benign causes

Physicians should have a broad differential diagnosis when encountering a patient with mesenteric panniculitis and not subject the patient routinely to undergo CT-guided biopsy to establish the diagnosis.

Physicians should also not order multiple radiological investigations and still a conservative approach is needed. The challenges faced are whether an aggressive approach of surgical intervention is needed. But it would be worth watching and a holistic approach of wait and watch is desired.

At the moment, as per our literature review, we cannot find a confirmed certain link between mesenteric panniculitis and subsequent malignancy.

The prevalence of MP appears to be much higher than previously reported, and the reason for this is likely the major technological evolution in imaging during the last decade. This high prevalence may explain the spontaneous association with the numerous and probably unrelated clinical situations found in the literature. Finally, the vast majority of cases are considered idiopathic, benign, and asymptomatic [8]. Furthermore, referring clinicians are often unfamiliar with MP and therefore look-up to the reporting radiologist for management guidance [25].

Lymph node size (>12 mm) and the absence of the fat ring sign were identified as predictors of subsequent diagnosis of malignancy in patients with MP. Identification of MP via imaging should prompt awareness for possible malignancy in these patients [19].

## Conclusions

High-quality research linking mesenteric panniculitis imaging features and subsequent malignancy is needed. The lack of consensus regarding the clinical significance of MP thus presents clinicians with a diagnostic dilemma, because MP is encountered frequently as an apparently incidental finding on cross-sectional imaging, usually abdomino-pelvic CT scan. There is no consensus on the treatment of MP. Treatment approaches in the literature mostly consist of supportive procedures intended to relieve the symptoms of MP. Physicians should apply holistic approach when they encounter mesenteric panniculitis which includes thorough physical examination, detailed history for red flag signs for malignancy and age-related appropriate screening for malignancy tailored to individual patients.
